# A comparison of the efficiency of five different commercial DNA extraction kits for extraction of DNA from faecal samples

**DOI:** 10.1016/j.mimet.2013.05.008

**Published:** 2013-08

**Authors:** Shantelle Claassen, Elloise du Toit, Mamadou Kaba, Clinton Moodley, Heather J. Zar, Mark P. Nicol

**Affiliations:** a Division of Medical Microbiology, Department of Clinical Laboratory Science, University of Cape Town, Cape Town, South Africa; b National Health Laboratory Service, National Institute for Communicable Diseases, Groote Schuur Hospital, Cape Town, South Africa; c Department of Paediatrics and Child Health, Red Cross War Memorial Children's Hospital, University of Cape Town, Cape Town, South Africa; d Institute of Infectious Disease and Molecular Medicine, University of Cape Town, Cape Town, South Africa

**Keywords:** Bacteria, DGGE, DNA extraction, Faeces, Real-time PCR, T-RFLP

## Abstract

Differences in the composition of the gut microbiota have been associated with a range of diseases using culture-independent methods. Reliable extraction of nucleic acid is a key step in identifying the composition of the faecal microbiota. Five widely used commercial deoxyribonucleic acid (DNA) extraction kits (QIAsymphony® Virus/Bacteria Midi Kit (kit QS), ZR Fecal DNA MiniPrep™ (kit Z), QIAamp® DNA Stool Mini Kit (kit QA), Ultraclean® Fecal DNA Isolation Kit (kit U) and PowerSoil® DNA Isolation Kit (kit P)) were evaluated, using human faecal samples. Yield, purity and integrity of total genomic DNA were compared spectrophotometrically and using gel electrophoresis. Three bacteria, commonly found in human faeces were quantified using real time polymerase chain reaction (qPCR) and total bacterial diversity was studied using denaturing gradient gel electrophoresis (DGGE) as well as terminal restriction fragment length polymorphism (T-RFLP). The measurements of DNA yield and purity exhibited variations between the five kits tested in this study. Automated kit QS exhibited the best quality and highest quantity of DNA. All kits were shown to be reproducible with CV values ≤ 0.46 for DNA extraction. qPCR results showed that all kits were uniformly efficient for extracting DNA from the selected target bacteria. DGGE and T-RFLP produced the highest diversity scores for DNA extracted using kit Z (H′ = 2.30 and 1.27) and kit QS (H′ = 2.16 and 0.94), which also extracted the highest DNA yields compared to the other kits assessed.

## Highlights

• Five commercial bacterial DNA extraction kits were compared on human faecal samples.
• Extraction efficiency and isolated bacterial diversity were evaluated.
• Quantitation by qPCR of targeted bacteria commonly found in human faeces
• Automated kit QS provided the highest DNA yield and best quality DNA.
• Automated kit QS extracted high bacterial diversity and provides practical advantages.

## 1 Introduction

Variations in the composition of the gut microbiota between individuals are increasingly recognized due to the development of culture-independent analytic techniques (Smith et al., 2011). These techniques have contributed to a wealth of studies over the past decade, investigating the human gut microbiota and its role in human health and disease (De Vos and De Vos, 2012, Maukonen et al., 2012). A key step to correctly identify the diversity of the gut microbiota from faecal samples is to obtain sufficient amounts of high quality genomic DNA, suitable for further analysis (Smith et al., 2011).

Deoxyribonucleic acid (DNA) extraction from faeces is challenging since several substances may be co-extracted, resulting in inhibitory effects on downstream applications (Persson et al., 2011, Wilson, 1997). Other challenges encountered when extracting DNA from faeces include incomplete cell lysis and shearing of DNA which may also affect downstream processing (Ariefdjohan et al., 2010, McOrist et al., 2002). Extracted genomic DNA needs to equally represent all microbial communities present within the sample in order to effectively characterize the microbiota present (Yuan et al., 2012). This poses further challenges as the human gastro-intestinal tract (GIT) is colonized with bacteria which have varying cell wall structures and compositions, making some species more difficult to lyse than others, such as those belonging to the genera Bifidobacteria and Lactobacillus (Maukonen et al., 2012).

This study aimed to compare the relative efficacy of extracting bacterial genomic DNA from human faecal samples using five commercial DNA extraction kits. The DNA extraction kits were evaluated based on their ability to efficiently lyse bacterial cells, cause minimal DNA shearing, produce reproducible results and ensure broad-range representation of bacterial diversity. Extraction efficiency was assessed by quantitative real time polymerase chain reaction (qPCR) and fingerprinting methods using denaturing gradient gel electrophoresis (DGGE) and terminal restriction fragment length polymorphism (T-RFLP). Selection of kits evaluated during this study was based on their commercial preference, availability and ease of use (Ariefdjohan et al., 2010, Bahl et al., 2012, Holland et al., 2000, Leite et al., 2013, Li et al., 2003, McOrist et al., 2002, Stauffer et al., 2008).

## 2 Materials and methods

### 2.1 Subjects and faecal sample collection

Faecal samples were collected in sterile containers from four infants under the age of two years and four adults, after obtaining consent from the healthy individuals or their guardians. Immediately after collection, samples were transported on ice and stored at − 70 °C prior to processing. This study was approved by the Research Ethics Committee of the University of Cape Town, South Africa.

### 2.2 DNA extraction from faecal samples

DNA was extracted in triplicate from each faecal sample using the five commercial kits (Table 1) according to their respective manufacturer's instructions, with slight modifications. Briefly, genomic DNA was extracted using two starting amounts of faecal material, 100 mg and 200 mg. Mechanical cell lysis (bead-beating) was carried out for all kits at 50 Hz for 5 min using the TissueLyser LT™ (Qiagen, FRITSCH GmbH, Idar-Oberstein, Germany), with the exception of QIAamp® DNA Stool Mini Kit (kit QA), seeing that a mechanical cell lysis step is not recommended by the manufacturer.

**Table 1:** The five commercial DNA extraction and purification kits used in this study.

Full name of the kit	Manufacturer details	Kit name abbreviation	Recommended faecal starting amount (mg)	Extraction method	Elution volume (μl)	Estimated price per kit (June 2012, ZAR)
QIAsymphony® Virus/Bacteria Midi Kit	Qiagen, Hilden, Germany	QS	Not specified	Automated	60, 85, 110^a^	4731
ZR Fecal DNA MiniPrep™	Zymo Research Corp., Irvine, USA	Z	150	Manual	100	2128
QIAamp® DNA Stool Mini Kit	Qiagen, Valencia, CA, USA	QA	180–220	Manual	200	2297
Ultraclean® Fecal DNA Isolation Kit	MoBio Laboratories Inc., Carlsbad, USA	U	250	Manual	50	2530
PowerSoil® DNA Isolation Kit	MoBio Laboratories Inc., Carlsbad, USA	P	250	Manual	100	2824

a Kit QS provides the option of three elution volumes (µl).

Automated extraction using the QIAsymphony® Virus/Bacteria Midi Kit (kit QS) incorporated an “off-board lysis” step using 750 μl lysis buffer from ZR Fecal DNA MiniPrep™ kit (kit Z) combined with mechanical cell lysis. The lysate was centrifuged at 10 000 rpm for 1 min, and 300 μl of the resulting clarified supernatant was used for the DNA extraction on the QIAsymphony® SP instrument (Qiagen, Hombrechtikon, Switzerland) as recommended by the manufacturer.

In order to ensure complete flow-through of supernatant during step 4 for kit Z, an additional centrifugation step at 14 000 rpm for 1 min was added.

Homogenisation of faecal samples, in Buffer ASL, for kit QA was performed using the TissueLyser LT™ (Qiagen, FRITSCH GmbH, Idar-Oberstein, Germany) at 50 Hz for 1 min. The optional heating of the faecal lysate at 95 °C (in step 4) was used instead of 75 °C. When required, the alternate RNase treatment, as recommended by the manufacturer was used. Extracted DNA was treated with 3.0 μg of RNase A (Sigma-Aldrich, Carlsbad, United States of America). Because the manufacturer's protocol did not include a subsequent clean-up step, in order to remove the degraded RNA and enzyme from the treated DNA, the wash steps in the protocol were performed from step 11, excluding the incubation step at step 12.

Ultraclean® Fecal DNA Isolation Kit (kit U) and PowerSoil® DNA Isolation Kit (kit P) extractions included the alternate lysis step, as per manufacturer's recommendations, which replaced step 5 for kit P and step 6 for kit U.

All extracted DNA was eluted in 50 μl of distilled water (H_2_O), except the automated kit QS, where the minimum elution volume allowed by the supplier was set at 60 μl using Buffer AVE.

### 2.3 Quality and quantity of DNA extracted from faecal sample

The DNA yield (ng) and purity (absorbance ratio at 260/280) of extracted genomic DNA were determined spectrophotometrically using the Nano Drop® ND-1000 (Nanodrop Technologies Inc., Wilmington, United States of America), where pure DNA is defined as having a 260/280 absorbance ratio ranging between 1.7 and 2.0 (Chen et al., 2010). The integrity of genomic DNA was determined by visualising approximately 200 ng of DNA on a 1% agarose gel (w/v) containing 0.25 μg/μl of ethidium bromide (EtBr), run in 1 × Tris-EDTA buffer at 100 V.

### 2.4 Evaluation of DNA extraction methods by real-time PCR

#### 2.4.1 Generation of a standard curve with genomic DNA

In order to assess the performance of commercial DNA extraction kits for DNA isolation from bacteria with varying cell wall structures and composition, standard curves were constructed using three control bacterial strains. *Bacteroides fragilis* 638R (donated by Prof. Abratt, Department of Molecular and Cell Biology, University of Cape Town, South Africa) and *Bifidobacterium longum* spp. *longum* JCM (Japan Collection of Microorganisms) 1217, were cultured on Brucella agar in a carbon dioxide (CO_2_) chamber (genebox anaer [Biomerieux]) under atmospheric conditions (85% nitrogen, 10% CO_2_ and 5% hydrogen) at 37 °C. *Escherichia coli* ATCC (American Type Culture Collection) 25922 was cultured aerobically on Mueller-Hinton agar at 37 °C.

Overnight cultures of *B. fragilis* and *B. longum* were washed off twice from the Brucella agar using 1 ml LB Broth during each wash, followed by centrifugation at 10 000 rpm for 1 min. The supernatant was discarded and cell pellets were re-suspended in 100 μl pre-lysis buffer (Magwira et al., 2012) (20 mM Tris–HCl (pH 8.0), 2 mM EDTA (pH 8.0), 1.2% (v/v) Triton X-114, 20 mg/ml lysozyme), and incubated at 37 °C for 30 min with agitation (300 rpm) (Thermomixer® Compact [Eppendorf AG, Hamburg, Germany]). Proteinase K (20 μg) (Sigma-Aldrich, Carlsbad, United States of America) was subsequently added, and the reaction incubated at 37 °C for 30 min, with agitation. Genomic DNA was then extracted from the pre-lysed bacterial strains using the kit Z protocol (Zymo Research Corp., Irvine, California, United States of America), by omitting step 11 which is specific for DNA extraction directly from faecal samples. DNA from *E. coli* was extracted using kit Z protocol after washing colonies from Mueller Hinton agar, with step 11 omitted. To quantify DNA extracted from faecal samples, standard curves were constructed using 10-fold serial dilutions of known concentrations of genomic DNA extracted from the three control strains.

#### 2.4.2 Quantification of target bacteria from faecal samples

Quantitative real time PCR was performed in duplicate for each of the triplicate DNA extractions using a CFX96™ Real-Time System (Bio-Rad Laboratories Inc., Hercules, CA, United States of America). Distilled H_2_O was used as a no template control. DNA extracted from the control bacterial strains (at a concentration of 150 μg) served as positive controls. The PCR reaction mixture (in a final volume of 25 μl) consisted of 1 × SensiFAST™ Probe No-ROX (Bioline, London, United Kingdom), 0.24 μM of each primer, 0.08 μM of probe (Inqaba Biotec, Pretoria, South Africa) and 15 ng of respective template DNA from faecal samples. All PCR oligonucleotides used and amplification conditions are listed in Table 2.

**Table 2:**
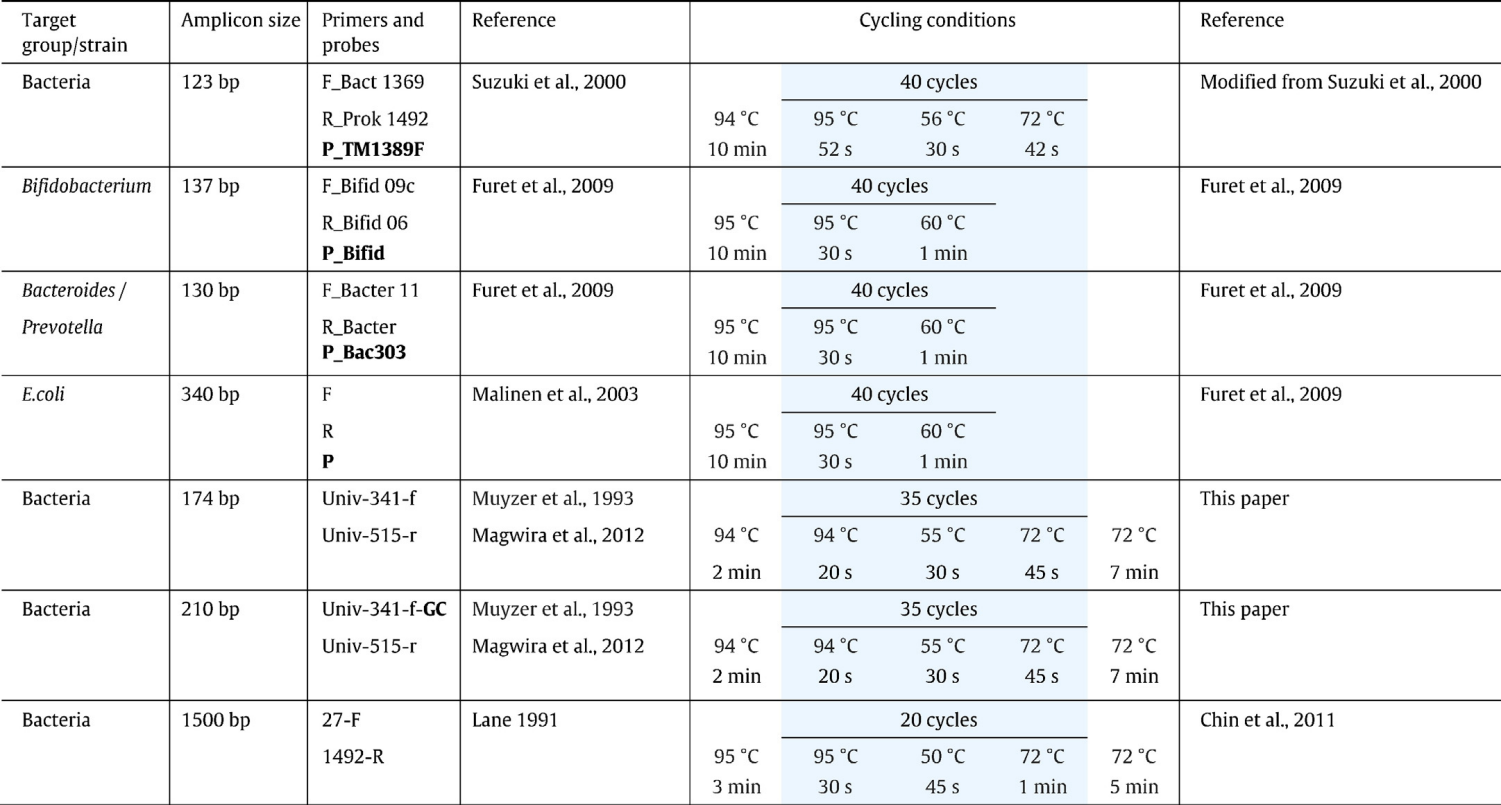
Primers and probes and cycling conditions. Suzuki et al., 2000, Furet et al., 2009, Malinen et al., 2003, Muyzer et al., 1993, Magwira et al., 2012, Lane, 1991, Chin et al., 2011

GC clamp and probe sequences are in boldface.

All oligonucleotide probes were labelled with 5′-reporter dye 6-carboxyfluorescein (6-FAM).

All oligonucleotide probes were labelled with 3’-quencher dye dimethylaminoazosulfonic acid (DABSYL).

### 2.5 Evaluation of DNA extraction efficiencies by fingerprinting techniques

#### 2.5.1 DGGE analysis of 16S rDNA amplicons

Two rounds of PCR were performed targeting bacterial 16S rDNA genes. The first round PCR reaction mixture amplified the 16S rDNA genes and was performed in a final volume of 25 μl containing 90 ng of DNA template (triplicate extractions pooled), 1 × ReadyMix (KAPA Biosystems, Cape Town, South Africa), 0.5 μM of each primer Univ-341-f and Univ-515-r (Table 2). The second round of the PCR added a GC-clamp at the 5′-end of the forward primer Univ-341-f (Muyzer et al., 1993). This PCR was performed in a final volume of 25 μl, containing 2 μl of the first round PCR product as template, 0.5 μM of each primer Univ-341-f-GC and Univ-515-r (Table 2) and 1 × ReadyMix (KAPA Biosystems, Cape Town, South Africa). Amplification conditions were the same for both rounds of PCR and are shown in Table 2.

DGGE was performed as previously described by Muyzer et al. (1993), with the following modifications. After polymerisation of denaturing gels (Vael et al., 2011), PCR fragments were separated using a denaturing gradient of 45% to 65% by loading 10 μl of the second round PCR reaction mix, together with 10 μl of 6 × loading dye into the well. Electrophoresis was carried out using the DCode™ Universal Mutation Detection System (Bio-Rad Laboratories Inc., Hercules, CA, United States of America) at 180 V for 10 min, followed by constant voltage (71 V) and temperature (60 °C) for 15 h 30 min. Gels were stained with EtBr (25 μl of 10 mg/ml EtBr in 250 ml 1 × TAE buffer) for 30 min, followed by de-staining with fresh 1 × TAE buffer for 20 min. DGGE banding patterns were visualised using ChemiDoc™ XRS^+^ with Image Lab™ software (Bio-Rad Laboratories Inc., Hercules, CA, United States of America). Quantity-One® software version 4.5.2 (Bio-Rad Laboratories Inc., Hercules, CA, United States of America) was used to evaluate the diversity index of each lane. The bacterial community was determined using the Shannon–Wiener index of diversity (H′ index) as previously described (Gafan et al., 2005).

#### 2.5.2 T-RFLP analysis of 16S rDNA amplicons

Bacterial 16S rDNA genes were targeted using the primer set 27-F (5′-FAM) and 1492-R (Table 2). The PCR was carried out in a reaction mixture of 50 μl containing 60 ng of DNA template (triplicate extractions pooled), 1 × ReadyMix (KAPA Biosystems, Cape Town, South Africa) and 0.5 μM of each primer (Inqaba Biotec, Pretoria, South Africa). The thermal cycling conditions are described in Table 2. The PCR products were purified using the QIAquick® PCR Purification Kit (Qiagen, Hilden, Germany), without adding pH indicator to Buffer PB. DNA was eluted in 50 μl of distilled H_2_O.

In order to select the appropriate enzyme resulting in the highest number of terminal restriction fragment (T-RF) peaks, five commonly used restriction endonucleases (*Alu*I, *Hae*III, *Hha*I, *Msp*I and *Rsa*I [Thermo Fisher Scientific, Inc., Waltham, United States of America]) were evaluated on one randomly selected sample. The reaction mixture for each enzyme used contained 10 μl of purified PCR product, 1 μl restriction enzyme, 2 μl of 0.33 × buffer and 17 μl of distilled H_2_O in a final volume of 30 μl. The digestion of purified amplicons was performed as recommended by the manufacturer for each enzyme. Capillary electrophoresis was performed on an ABI3130xl (Applied Biosystems, Johannesburg, South Africa). T-RFLP profiles from each digested product were analysed using Peak Scanner software version 1.0, with the internal size standard set as GS500. T-RFs with a peak height of less than 25 fluorescence units were excluded from analysis, this in order to exclude background noise (Sakamoto et al., 2003). The analysis of the T-RFs was carried out using the R software version 2.15.1 (R Foundation for Statistical Computing, Vienna, Austria) and Primer 6 software version 6.1.11 (PRIMER-E Ltd., Plymouth, United Kingdom). The H′ index was calculated using Primer 6 software version 6.1.11.

### 2.6 Statistical analysis

All statistical analyses were performed using GraphPad Prism version 6.02 (GraphPad Software, Inc., California, United States of America). Normality was tested for all datasets using the D'Agostino Pearson omnibus normality test. Pairwise comparisons were conducted, as appropriate, using two-tailed unpaired t tests. Parametric data was analysed using the *t* test with Welch's correction (not assuming equal standard deviations (SDs)). Non-parametric data was analysed using the Mann–Whitney test. Multiple comparisons were conducted using one-way analysis of variance (for parametric data) or Kruskall–Wallis (for non-parametric data) tests. Correction for multiple comparisons was performed using the Holm–Sidak test for parametric data and Dunn's test for non-parametric data. A p-value less than 0.05 was considered as statistically significant.

## 3 Results

### 3.1 Evaluation of genomic DNA extracted from faecal samples

#### 3.1.1 DNA yield and purity

For extractions from 100 mg of faecal starting material, kit QA yielded, before RNase treatment and clean-up, a markedly higher median DNA yield (15717 ng) for all kits evaluated and had significantly higher DNA yields than kits U (694.2 ng; p < 0.0001) and P (1318 ng; p = 0.0018). The DNA yield was significantly higher for kit QS (8180 ng) when compared to kit U (694.2 ng; p = 0.0079). With regard to DNA purity (as expressed by the 260/280 absorbance ratio), kit QA (2.095) was less pure compared to kits Z (1.573; p < 0.0001), U (1.742; p = 0.0096) and P (1.658; p = 0.0008) (data not shown).

As for the 100 mg of faecal starting material, the extractions from 200 mg also revealed that kit QA, before RNase treatment and clean-up, had the highest median DNA yield (26754 ng) for all kits evaluated. It was significantly higher when compared to kits U (1504 ng; p < 0.0001) and P (1988 ng; p = 0.0002). With regard to DNA purity (absorbance 260/280), kit QA (2.095) was again less pure than kits Z (1.678; p = 0.0009), U (1.627; p = 0.0032) and P (1.617; p = 0.0011) (data not shown).

**Fig. 1:**
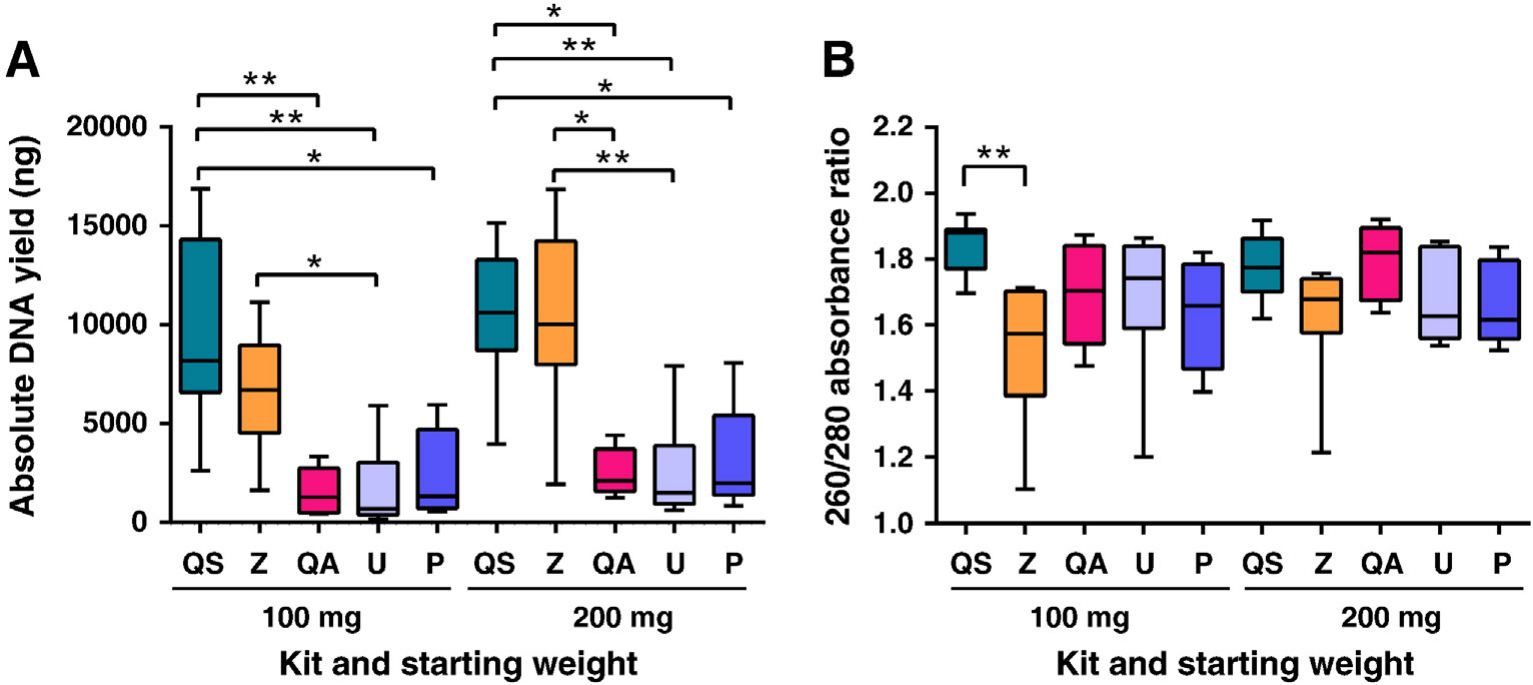
DNA yield and purity extracted from faecal specimens using five commercial kits. DNA was extracted in triplicate from two starting weights (100 mg and 200 mg) of faeces per sample (n = 8 samples per kit). Median values are indicated by the line within the box plot. The box extends from the 25th to 75th percentiles and whiskers indicate the minimum and maximum values. * p < 0.05; ** p ≤ 0.01. A) Boxplot showing DNA yields obtained for all five kits evaluated. Kit QA values after RNA degradation and clean-up. B) Figure showing absorbance ratios at 260/280 for all five kits assessed. Kit QA values after RNA degradation. QS: QIAsymphony® Virus/Bacteria Mini Kit (Qiagen); Z: ZR Fecal DNA Isolation Kit™ (Zymo Research); QA: QIAamp® DNA Stool Mini Kit (Qiagen); U: Ultraclean® Fecal DNA Isolation Kit (Mobio); P: PowerSoil® DNA Isolation Kit (Mobio).

Of interest, for DNA extracted using kit QA, gel electrophoresis showed the presence of large amounts of RNA (data not shown). After the addition of RNase A, as recommended by the manufacturer, no significant difference was found when comparing DNA yields for kit QA before and after RNase treatment (data not shown). With regard to DNA purity following the RNase treatment, absorbance ratios at 260/280 decreased significantly (p < 0.0001) for both 100 mg (1.693) and 200 mg (1.79) faecal material (Fig. 1B). Subsequently, kit QS now exhibited significantly purer 260/280 absorbance ratios (p = 0.0042) when compared to kit Z for 100 mg of faecal starting material (Fig. 1B).

Even though no difference was observed for kit QA with regard to DNA yield after RNase treatment, there was a significant change in the DNA yield following an RNA clean-up step (Fig. 1A), for both 100 mg (1294 ng; p < 0.0001) and 200 mg (2128 ng; p < 0.0001) of faecal material. This resulted in kits QS and Z exhibiting significantly higher DNA yields when compared to the other kits evaluated (Fig. 1A). Kit QS extracted the highest DNA yields for both 100 mg and 200 mg faecal starting material, but was not significantly higher than kit Z (Fig. 1A).

Our study showed that 200 mg starting amount of faeces yielded more DNA when compared to 100 mg (Fig. 1A), although not statistically significantly. No significant differences were observed between the two starting amounts in terms of the DNA purity (Fig. 1B). Therefore, in order to ensure homogeneity, DNA was extracted from 200 mg starting material for all further processing for all kits.

#### 3.1.2 Extraction reproducibility and DNA integrity analysis

Extractions were shown to be reproducible for all kits, with median coefficient of variation (CV) values being < 1 (kit QS = 0.14, kit Z = 0.19, kit QA = 0.46, kit U = 0.20, kit P = 0.20). The overall integrity of DNA extracted using all five kits was of a high standard as no shearing was observed on gel electrophoresis (data not shown).

### 3.2 Quantitative differences in extracting DNA from different bacteria

**Fig. 2:**
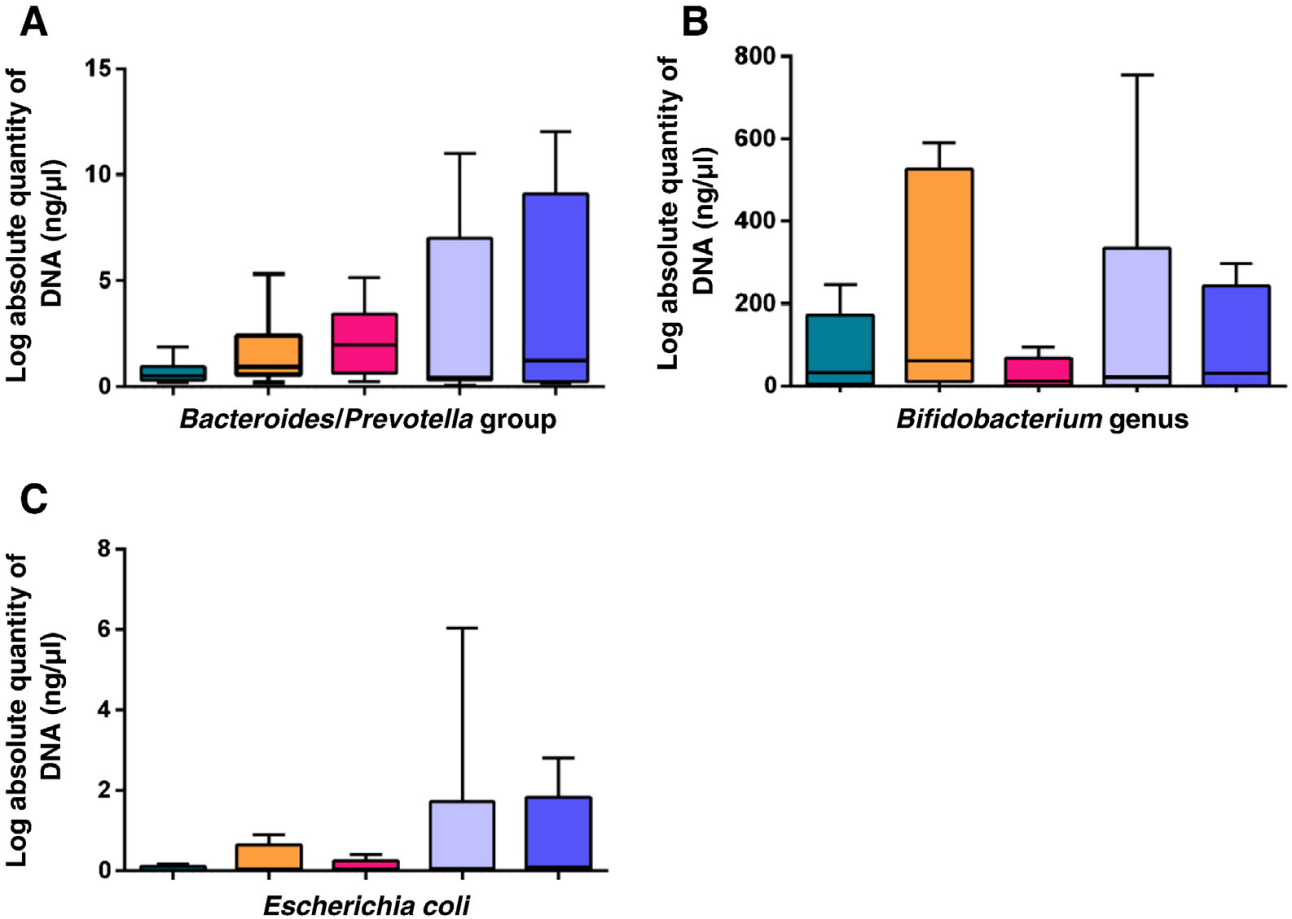
Absolute DNA concentration for three targeted bacterial strains/groups using template DNA extracted with five commercial kits. DNA was extracted in triplicate from 200 mg of faeces per sample (n = 8 samples per kit). Median values are indicated by the line within the box plot. The box extends from the 25th to 75th percentiles and whiskers indicate the minimum and maximum values. A) Boxplot showing absolute DNA concentration extracted for *Bacteroides*/*Prevotella* group; B) *Bifidobacterium* genus; and C) *Escherichia coli*. QS: QIAsymphony® Virus/Bacteria Mini Kit (Qiagen); Z: ZR Fecal DNA Isolation Kit™ (Zymo Research); QA: QIAamp® DNA Stool Mini Kit (Qiagen); U: Ultraclean® Fecal DNA Isolation Kit (Mobio); P: PowerSoil® DNA Isolation Kit (Mobio).

The qPCR targeting the three bacteria commonly found in faeces showed no consistent differences in amplification of DNA for these specific bacteria as demonstrated by qPCR testing using DNA extracted by the various kits (Fig. 2). All kits were reproducible for extracting DNA from the targeted bacteria by qPCR, as demonstrated by median CV values < 1 (kit QS = 0.29, kit Z = 0.34, kit QA = 0.46, kit U = 0.35, kit P = 0.29).

### 3.3 Bacterial diversity analysis by DGGE

**Fig. 3:**
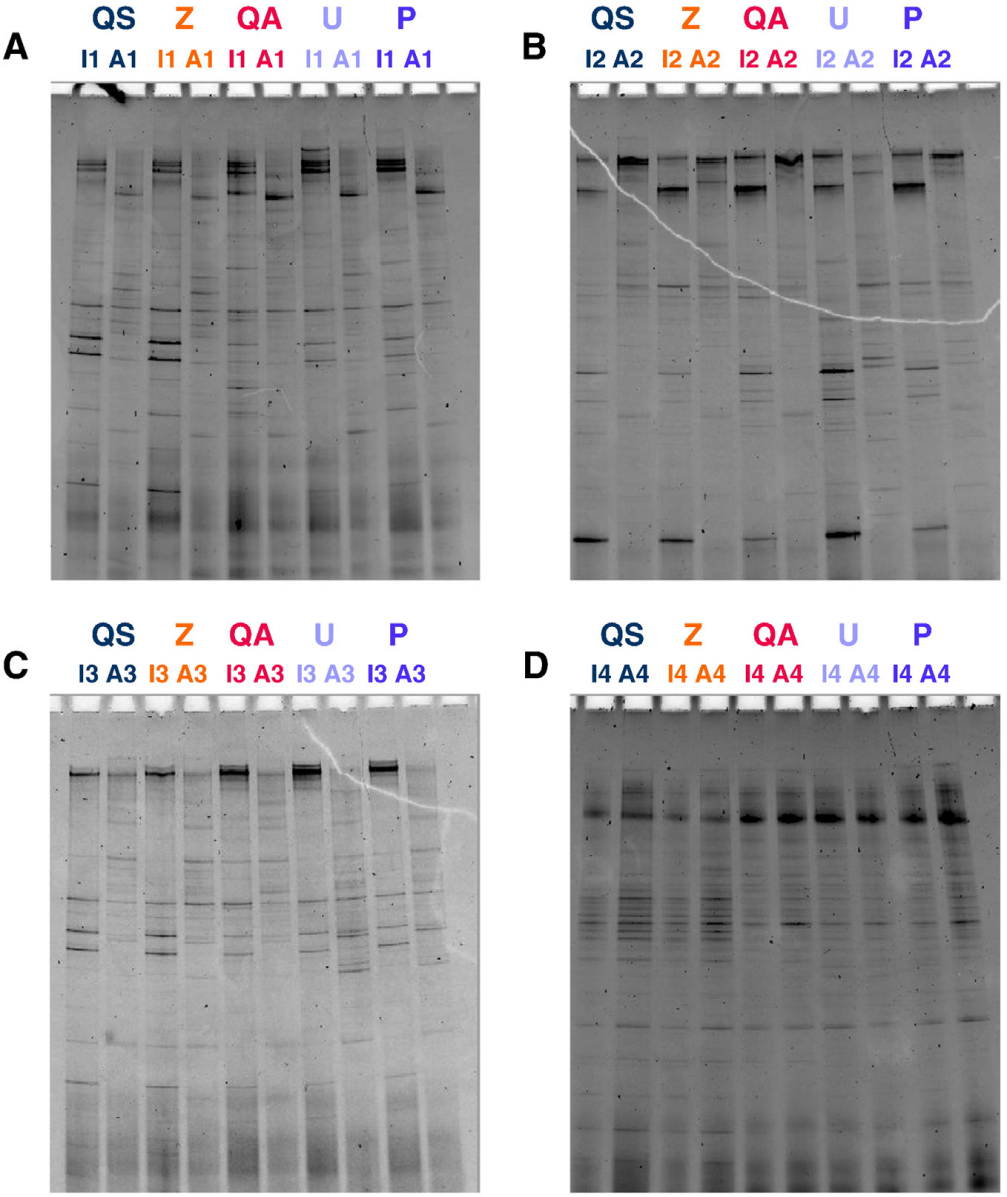
DGGE profiles of the 16S rDNA genes from DNA extracted using five commercial kits. DNA was extracted in triplicate from 200 mg of faeces per sample (n = 8 samples per kit) and run on a 45–65% DGGE gel gradient. A); Infant 1 (I1) and Adult 1 (A1); B); Infant 2 (I2) and Adult 2 (A2); C); Infant 3 (I3) and Adult 3 (A3); D); Infant 4 (I4) and Adult 4 (A4). QS: QIAsymphony® Virus/Bacteria Mini Kit (Qiagen); Z: ZR Fecal DNA Isolation Kit™ (Zymo Research); QA: QIAamp® DNA Stool Mini Kit (Qiagen); U: Ultraclean® Fecal DNA Isolation Kit (Mobio); P: PowerSoil® DNA Isolation Kit (Mobio).

**Fig. 4:**
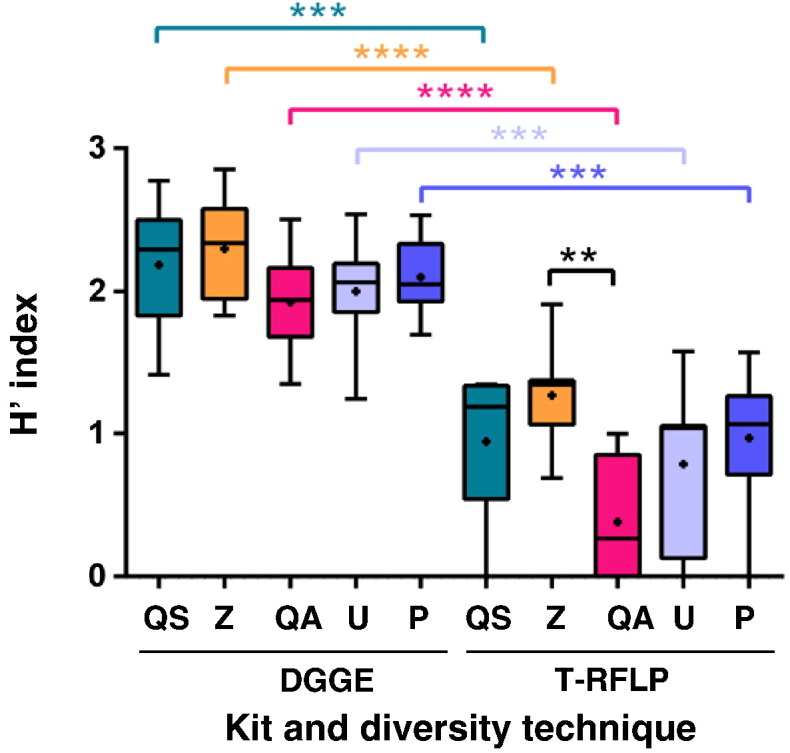
Bacterial diversity obtained from DNA extracted from faecal specimens using five commercial kits. Diversity (H′ indices) was determined from DNA extracted in triplicate from 200 mg of faeces per sample (n = 8 samples per kit), using two fingerprinting techniques. Mean H′ indices are indicated with a “+” within each box-and-whisker plot. Median values are indicated by the line within the box plot. The box extends from the 25th to 75th percentiles and the whiskers indicated the minimum and maximum values obtained. **p ≤ 0.01; ***p ≤ 0.001; **** p ≤ 0.0001 QS: QIAsymphony® Virus/Bacteria Mini Kit (Qiagen); Z: ZR Fecal DNA Isolation Kit™ (Zymo Research); QA: QIAamp® DNA Stool Mini Kit (Qiagen); U: Ultraclean® Fecal DNA Isolation Kit (Mobio); P: PowerSoil® DNA Isolation Kit (Mobio).

The efficiency of DNA extraction from the eight faecal samples using the five commercial kits was similar, as indicated by the representative banding patterns in Fig. 3. The highest mean H′ index was obtained for kit Z (H′ = 2.30), however there was no statistically significant difference between the five kits assessed (Fig. 4). The reproducibility of this technique was demonstrated by repeating both amplification and DGGE using randomly selected samples from two participants (Infant 2 and Adult 2) which resulted in a mean change in H′ index of only 0.02 (data not shown).

### 3.4 Bacterial diversity analysis by T-RFLP

After comparing the profiles generated by five commonly used restriction enzymes, the enzymes *Hha*I and *Msp*I provided the highest number of peaks on resulting electropherograms (data not shown). These two restriction enzymes were subsequently used for individual digestion of all samples analysed. When comparing mean H′ indices obtained from these two restriction enzymes, *Msp*I (H′ = 0.87) gave higher H′ indices than *Hha*I (H′ = 0.82) for all kits, although not significantly (data not shown).

### 3.5 Comparison of DGGE and T-RFLP data

The results of the comparison of DGGE and T-RFLP are shown in Fig. 4. For both techniques, kit Z gave the highest mean H′ index (H′ = 2.30 and 1.27, respectively), followed by kit QS (H′ = 2.19 and 0.94, respectively), although there was no significant difference between these kits. T-RFLP analysis showed that kit Z had a significantly higher H′ index compared to kit QA (H′ = 0.38; p = 0.0059). All five kits assessed in this study produced significantly higher H′ indices from DGGE analysis when compared to T-RFLP.

## 4 Discussion

Over the past decade, the influence of the human gut microbiota on health and disease has been the focus of numerous studies (De Vos and De Vos, 2012, Fujimura et al., 2010), with faecal samples being widely used (Maukonen et al., 2012, Penders et al., 2007). Even though faeces may only partly represent the complexity of the GIT microbiota (Hayashi et al., 2005), it is sampled non-invasively and easy to acquire at minimal cost (Dave et al., 2012). Despite sampling procedures not being complex, DNA extraction from faeces is challenging due to the variable consistency thereof which may affect the outcome of downstream processing (Holland et al., 2000). In addition, the vast bacterial diversity present results in different optimal DNA extraction methods for different cell wall structures and compositions (Maukonen et al., 2012). Therefore, faecal microbial community profiling studies require an optimised DNA extraction protocol ensuring efficient cell lysis, minimal DNA shearing and the removal of PCR inhibitors. In addition, it also needs to generate the best overall representation of the microbial community present (Maukonen et al., 2012, McOrist et al., 2002).

In our study, five commercial DNA extraction kits were selected for comparison based on their commercial preference, availability and ease of use (Ariefdjohan et al., 2010, Bahl et al., 2012, Holland et al., 2000, Li et al., 2003, McOrist et al., 2002, Stauffer et al., 2008). DNA extraction kits were evaluated using faecal samples collected from infants under the age of two years, as well as adults. These populations were selected to assess extraction efficiency from a broad range of microbial profiles, ranging from a highly variable and less stable microbial profile found in infants during the first year of life to a more stable profile typically found in adults (Palmer et al., 2007, Yatsunenko et al., 2012, Zoetendal et al., 1998). Faecal sample starting weights used in this study were selected in order to ensure homogeneity during the assessment of the five different kits. The faecal starting material recommended by the respective manufacturers of the five kits used in this study ranged from 150 - 250 mg (Table 1). We selected 100 mg and 200 mg starting weights of faecal material for comparison. The 100 mg starting weight was selected because a previous study showed that lower starting amounts of faecal material produced significantly higher DNA yields (Ariefdjohan et al., 2010), and 200 mg as it fell within the prescribed ranges of the kits. Since a study conducted by Ariefdjohan et al. (2010), showed that maximum DNA yields are obtained from using 10–50 mg of faecal starting material when compared to 100 and 200 mg, it might be interesting to compare the performance of DNA extraction kits evaluated in this study using a series of lower starting amounts of faecal material.

All kits evaluated were found to be reproducible (CV values < 1) in isolating bacterial DNA from faecal samples, with minimal DNA shearing. However DNA of poor purity have been obtained using kit QA without the RNase treatment and removal step, as observed by the 260/280 absorbance ratio and on the gel electrophoresis. This is consistent with what has been previously observed for kit QA, with 260/280 absorbance ratios reaching a high of 3.25 (Vanhoutte et al., 2004). Following the treatment with RNase A (as suggested by the manufacturer's protocol) the absorbance at 260/280 decreased significantly, but not the DNA yield. The manufacturer's protocol did not include a RNA clean-up step, resulting in fragmented RNA being measured during spectrophotometric analysis. In support of kit QA possibly extracting lower DNA yields than those resulting from spectrophotometrical analysis, Bahl et al. (2012) confirmed lower DNA yields for kit QA when compared to kit P. This might be explained by the fact that kit QA rely on chemical and heat lysis when compared to kits incorporating mechanical lysis Ariefdjohan et al. (2010).

Low DNA yields were obtained for kits U and P in our study, which is consistent with what has been found previously (Ariefdjohan et al., 2010, Scupham et al., 2007). This is of importance as the Human Microbiome Project (HMP) (McInnes and Cutting, 2010, Li et al., 2012) makes use of kit P (PowerSoil® DNA Isolation Kit) for isolating DNA from faecal samples. The HMP aims to provide resources for characterization of the human microbiota across multiple habitats and its association with human health and disease (McInnes and Cutting, 2010). Therefore, bias may be introduced in the HMP microbial profiles, as lower yields of genomic DNA has been shown to result in a less comprehensive reflection of the microbial community contained by the sample (Ariefdjohan et al., 2010).

A study conducted by Maukonen et al. (2012) showed that the microbial profiles from faecal samples are significantly affected by the commercial DNA extraction kits employed. Higher yields of genomic DNA allow for a more comprehensive reflection of the microbial community contained by the sample (Ariefdjohan et al., 2010). Therefore, an increase in DNA extraction efficiency also results in increased chances of detecting less-common species (Ariefdjohan et al., 2010). Our findings using DGGE and T-RFLP showed that kit Z gave the highest diversity followed by kit QS (Fig. 4), which are consistent with this argument since these were the kits that yielded the highest absolute DNA yields. Diversity obtained from kit QA was significantly lower than kit Z for T-RFLP analysis (p = 0.0059), supporting the confirmed lower DNA yields obtained after RNA clean-up. The lower diversities obtained for both DGGE and T-RFLP, from extracted DNA using kits QA and U are in agreement with a previous study comparing these kits to two other DNA extraction kits, FastDNA® SPIN Kit and FastDNA® SPIN Kit for Soil (Ariefdjohan et al., 2010). Our findings confirming the reproducibility of DGGE are in agreement with previous studies (Nicolaisen and Ramsing, 2002, Suchodolski et al., 2004). Regardless of the kit used, H′ indices for DGGE were significantly higher than those obtained from T-RFLP, with p values being ≤ 10^− 3^ for all five kits. This could be the result of the use of a single restriction enzyme and fluorescently-labelled primer in digestion reactions as opposed to a combination of enzymes and primers (Schütte et al., 2008).

## 5 Conclusions

The highest DNA yields were obtained for kits QS and Z, with kit QS also extracting the best quality of DNA. All five kits were uniformly efficient for extracting DNA from the three targeted bacteria, based on real time qPCR results. Using the fingerprinting techniques, kit Z and QS extracted the highest bacterial diversity. We strongly recommend the incorporation of the suggested RNase treatment for kit QA during the extraction protocol in order to remove RNA contamination and prevent misleading spectrophotometric readings. Finally, based on our findings here, automated kit QS which extracted the best quality and highest quantity of DNA and which also allows for the rapid processing of up to 96 samples per run, would be the recommended DNA extraction kit.

## Financial support

This study was funded by The Bill and Melinda Gates Foundation (Global Health Grant OPP1017641), Harry Crossley Research Fellowship and the University of Cape Town. The funders had no role in the study design, data collection and analysis, decision to publish, or preparation of the manuscript.

Mamadou Kaba is a recipient of Carnegie Corporation of New York (Unites States of America) Fellowship.

## Potential conflicts of interest

There is no potential conflict of interest to declare.
